# Editorial: Strategies for mitigating the transition period stress in dairy cattle

**DOI:** 10.3389/fvets.2023.1157526

**Published:** 2023-03-02

**Authors:** Mohanned Naif Alhussien, Ajay Kumar Dang, Dengpan Bu

**Affiliations:** ^1^Reproductive Biotechnology, School of Life Sciences Weihenstephan, Technical University of Munich, Freising, Germany; ^2^Lactation and Immuno-Physiology Laboratory, Indian Council of Agricultural Research (ICAR)-National Dairy Research Institute, Karnal, Haryana, India; ^3^State Key Laboratory of Animal Nutrition, Institute of Animal Sciences, Chinese Academy of Agricultural Sciences, Beijing, China

**Keywords:** dairy cattle, transition period, metabolic and immune dysfunctions, postpartum diseases, stress relieving strategies

The transition period is one of the most complicated physiological stages in the production cycle of dairy cows and their offspring. The major challenge after calving is the instability between body reserves and nutrient requirements for milk production. The abrupt increase in metabolic rate, associated with parturition and the onset of lactation, leads to a greater stress load that contributes to increased metabolic and infectious disease susceptibility (1). A comprehensive understanding of the epidemiology of various diseases associated with the transition period is crucial for identifying key risk factors and implementing strategies to mitigate them (2). Furthermore, investigating the metabolic, physiological, and immunological changes associated with the transition period is essential for the development and implementation of interventions that aim to reduce health disorders and improve the profitability of dairy animals (3). Therefore, the main objective of this Research Topic was to bring together the latest research, including management, therapeutic, and nutritional intervention strategies that minimize health complications associated with the transition period and promote the health and welfare of dairy cattle. Of particular interest are nutritional interventions that are central to strengthening immune response, reducing the incidence of various diseases associated with parturition, improving the quality of colostrum and milk, and subsequently promoting the growth and health of newborn calves.

This Research Topic presents novel and exciting feeding management as an intervention strategy to optimize the health and production of transition dairy cows. Three articles highlighted the importance of supplementing transition cows with rumen-protective amino acids such as lysine and methionine. Elsaadawy, Wu, Bu, et al. investigated the effects of supplying rumen-protected lysine (RPL) and methionine (RPM) to periparturient dairy cows on the efficiency of subsequent lactation. The study was conducted from 3 weeks before calving until the 150th day in milk, on four groups of cows (*n* = 25) receiving either a control diet, RPL, RPM, or a combination of both. The results demonstrated that the transition cows fed RPM and RPL had higher dry matter intake, milk production, milk protein yield, nitrogen efficiency, and improved fertility performance. In a subsequent article, Elsaadawy, Wu, Wang, et al. reported the average β-hydroxybutyrate concentrations during the 3 week post-calving period for cows that were supplied either RPL or RPM; they were at the lower threshold range of subclinical ketosis, while cows fed a combination were within the normal range of β-hydroxybutyrate concentrations. The authors suggest that supplementation of RPL and RPM to transition cows is a practical strategy for reducing negative energy balance and improving the metabolic adaptation of peripartum dairy cows, which is crucial for reducing the occurrence of postpartum diseases and enhancing milk yield and reproductive performance. Wang et al. also demonstrated that supplementing periparturient dairy cows with rumen-protected lysine or methionine, or a combination of both, improved the nutritional and immunological properties of colostrum and milk and increased passive immunity, calf health, and growth rate.

The supplementation of polyunsaturated fatty acids (PUFAs) is a crucial approach for mitigating the negative effects of negative energy balance and for improving the functions of the immune system in periparturient dairy cows, due to its anti-inflammatory, immunomodulatory, and antioxidant properties. Mezzetti et al. reported that the administration of an intravenous infusion of emulsified fish oil rich in long chained n3-PUFAs to early lactating cows resulted in enhanced milk production and reduced inflammatory and oxidative stress. Kim et al. investigated the dynamics of rumen pH and the composition of the rumen bacterial community in Holstein cows during the periparturient period. The study found that calving and postpartum dietary shifts did not provoke significant changes in the rumen bacterial community of cows during the transition period; however, dietary changes could activate the functional pathways of certain bacteria to meet energy demands and facilitate adaptation to a changed diet and the associated lower pH in the rumen.

In recent years, there has been a growing interest in the use of plant-based feed additives as a natural, multifunctional, and non-toxic approach to promote the health and production of dairy animals without the risks associated with the use of antibiotics. Cui et al. demonstrated that supplementation of a mixture of natural Chinese herbs to periparturient cows increased feed intake, milk production, and reduced concentrations of metabolites associated with a negative energy balance.

Finally, this Research Topic expands our knowledge on different strategies to mitigate transition stress in dairy cows and paves the way for further research in this exciting field. Despite the wide range of feeding strategies presented in these six articles, there are still areas of knowledge that remain to be explored. To optimize lactation and promote calf health, future research should focus on understanding the role of nutrition and management in improving the immunological, endocrinological, and biochemical functions of transition cows. Besides, using more efficient supplementation methods, such as rumen-protected amino acids, vitamins, and minerals, as well as implementing injection methods to provide and effectively improve the availability of essential nutrients is critical for enhancing the health and production of transition animals. Investigating the underlying molecular and cellular mechanisms of inflammatory and oxidative stress responses in transition dairy cows through the utilization of advanced techniques such as transcriptomics and proteomics is crucial for the identification of novel biomarkers. These can be utilized to predict and promote the health and production outcomes of transition dairy cows. Genomic studies on cattle and genetic selection of cows that are adapted to early lactation challenges could be a viable strategy for developing more resilient transition animals. Additionally, future studies should investigate the interactions between risk factors for various health complications that occur during the transition period, to develop more effective nutritional management strategies that prevent metabolic disorders, improve animal welfare, and increase farmer profitability.

## Author contributions

This Research Topic on management of Transition Period Stress was initially proposed and set up by MA. All editors worked together to establish the criteria for papers to be included in this Research Topic, and each manuscript was reviewed by the editorial group and reviewers. All editors contributed their thoughts and revisions to shape the published document.
